# Hyper-dominance of the left anterior descending artery—a large territory

**DOI:** 10.1259/bjrcr.20200069

**Published:** 2020-10-22

**Authors:** Gautam Sen, Alice Veitch, Manas Sinha

**Affiliations:** 1 Department of Cardiology, Salisbury Hospital, Odstock Rd, Salisbury, SP2 8BJ, United Kingdom; 2 Department of Radiology, Salisbury Hospital, Odstock Rd, Salisbury, SP2 8BJ, United Kingdom

## Abstract

Coronary artery anomalies are rare and a potential cause of significant morbidity and mortality. A hyper-dominant left anterior descending artery is extremely rare with only 17 cases reported in the literature. Occlusion of a hyper-dominant left anterior descending artery can cause a massive myocardial infarction affecting a large myocardial territory and therefore clinicians should be aware of its importance.

## Case presentation

A 38-year-old gentleman was seen in cardiology clinic, presenting with a syncopal episode whilst he was running. He had not complained of any breathlessness or chest pain. His previous medical history included hypertension for which he took amlodipine. There was no family history of cardiac disease. A 12-lead electrocardiogram showed normal sinus rhythm and a transthoracic echocardiogram showed normal biventricular systolic function with normal valvular function. He had a normal exercise stress test and had a reveal device implanted to look for any sinister arrhythmias. For completeness, he had a CT coronary angiogram (CTCA) to exclude coronary artery disease and anomalous coronary arteries.

**Figure 1.**
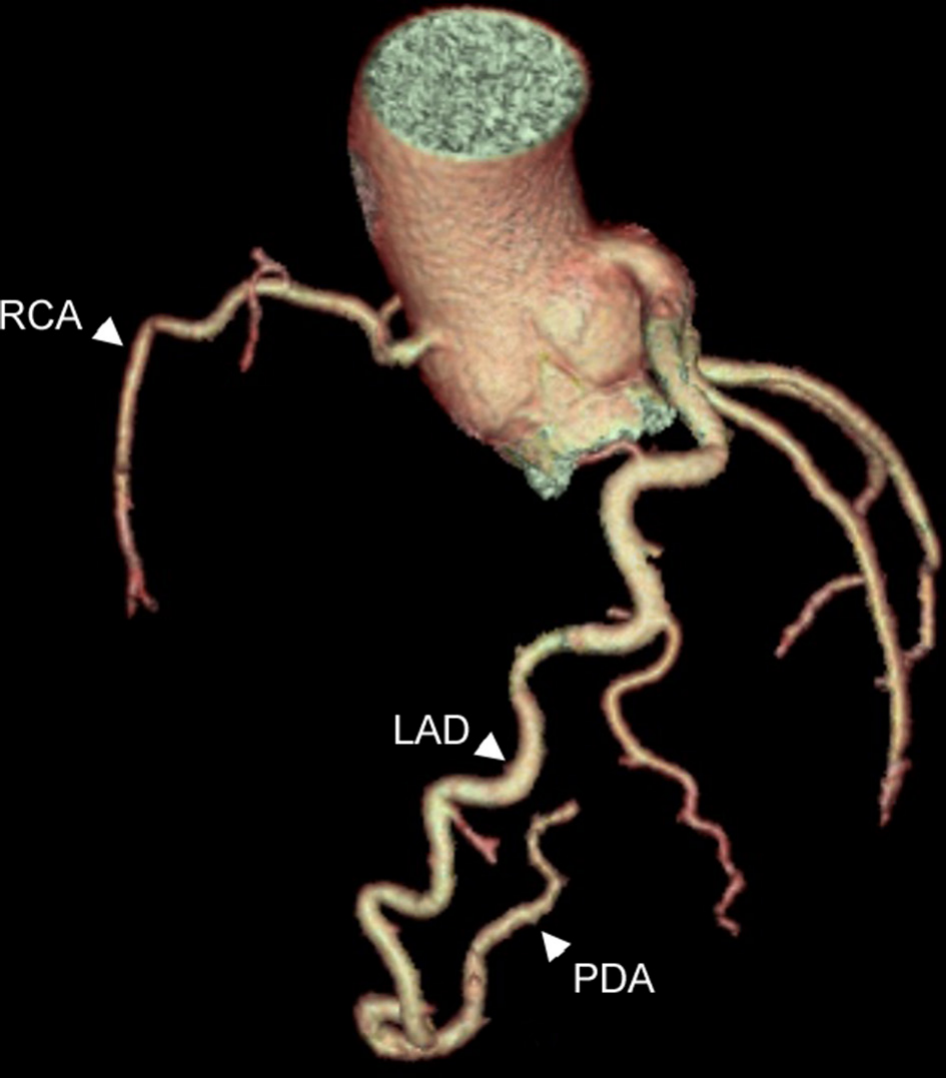
3D-reformat of CT coronary angiogram Figure showing a very tortuous and large calibre left anterior descending artery (LAD) located in its normal territory, but with the anomalous posterior descending artery (PDA) seen arising from it distally. The right coronary artery (RCA) is small and non-dominant.

**Figure 2.**
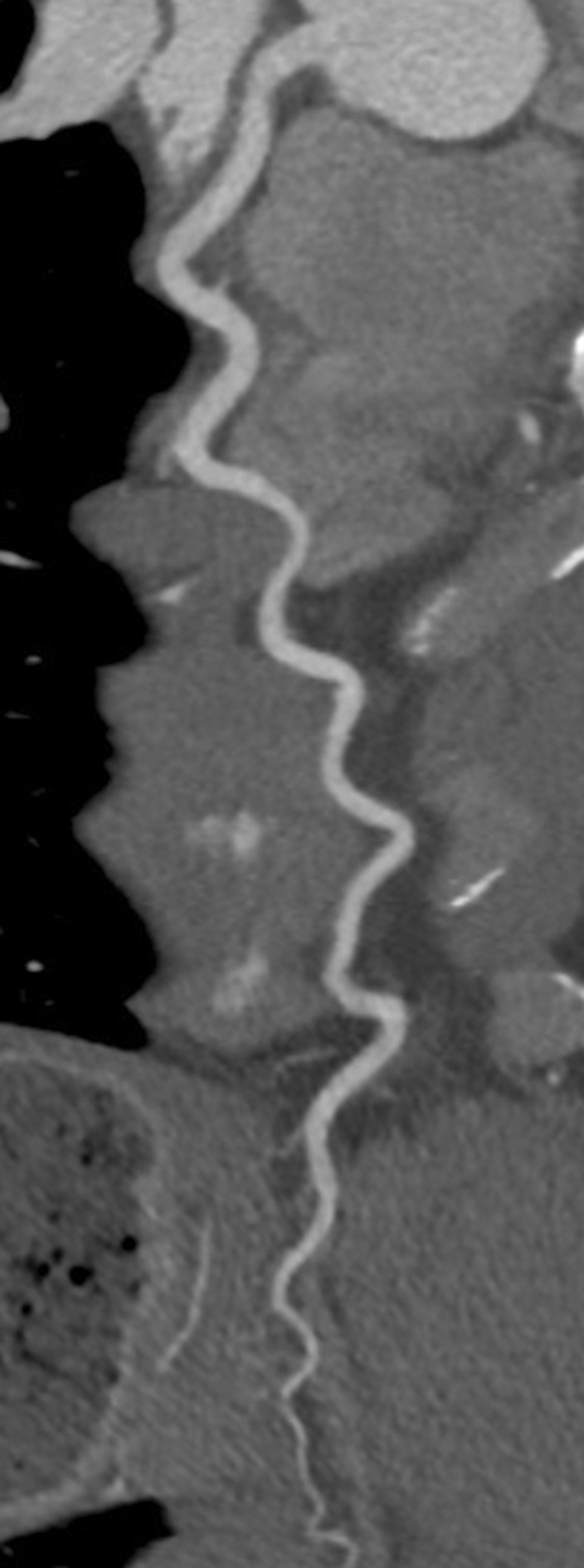
Multiplanar curved reformat of CT coronary angiogram Figure showing the large calibre left anterior descending artery (LAD) with a tortuous course and continuing as the posterior descending artery (PDA) distally. No significant coronary artery disease was identified.

**Figure 3.**
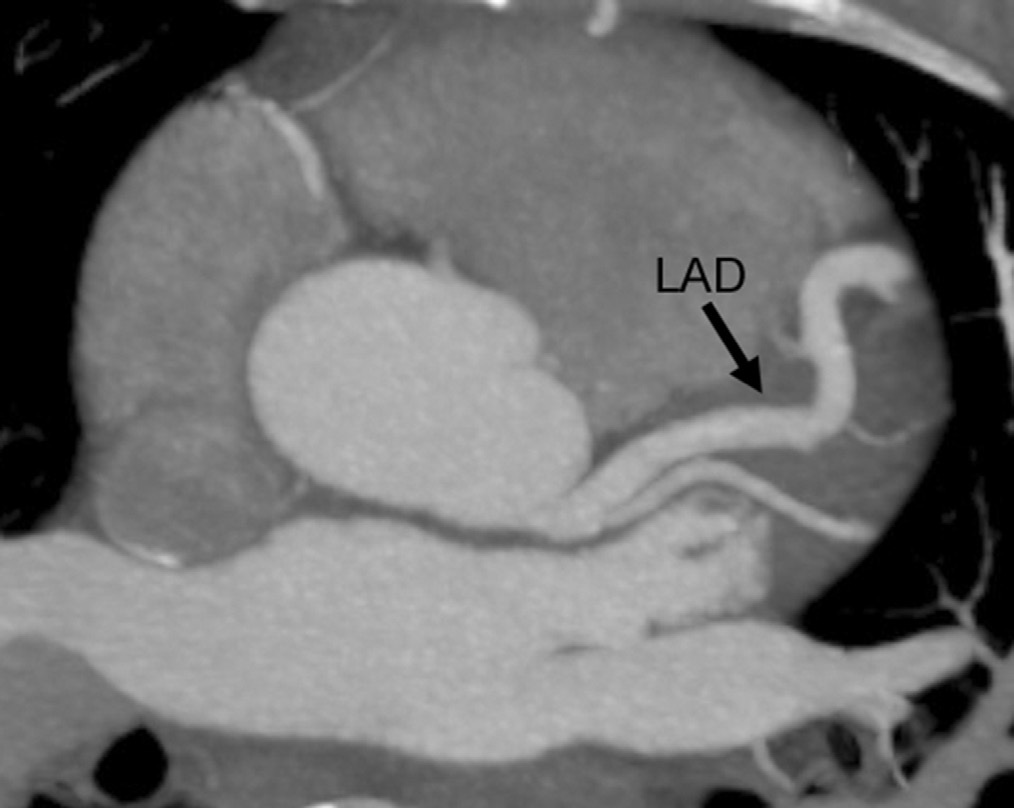
CT coronary angiogram superior axial maximum intensity projection Figure showing a large and tortuous left anterior descending artery (LAD) originating from the left coronary cusp and situated in its usual anatomical location.

**Figure 4.**
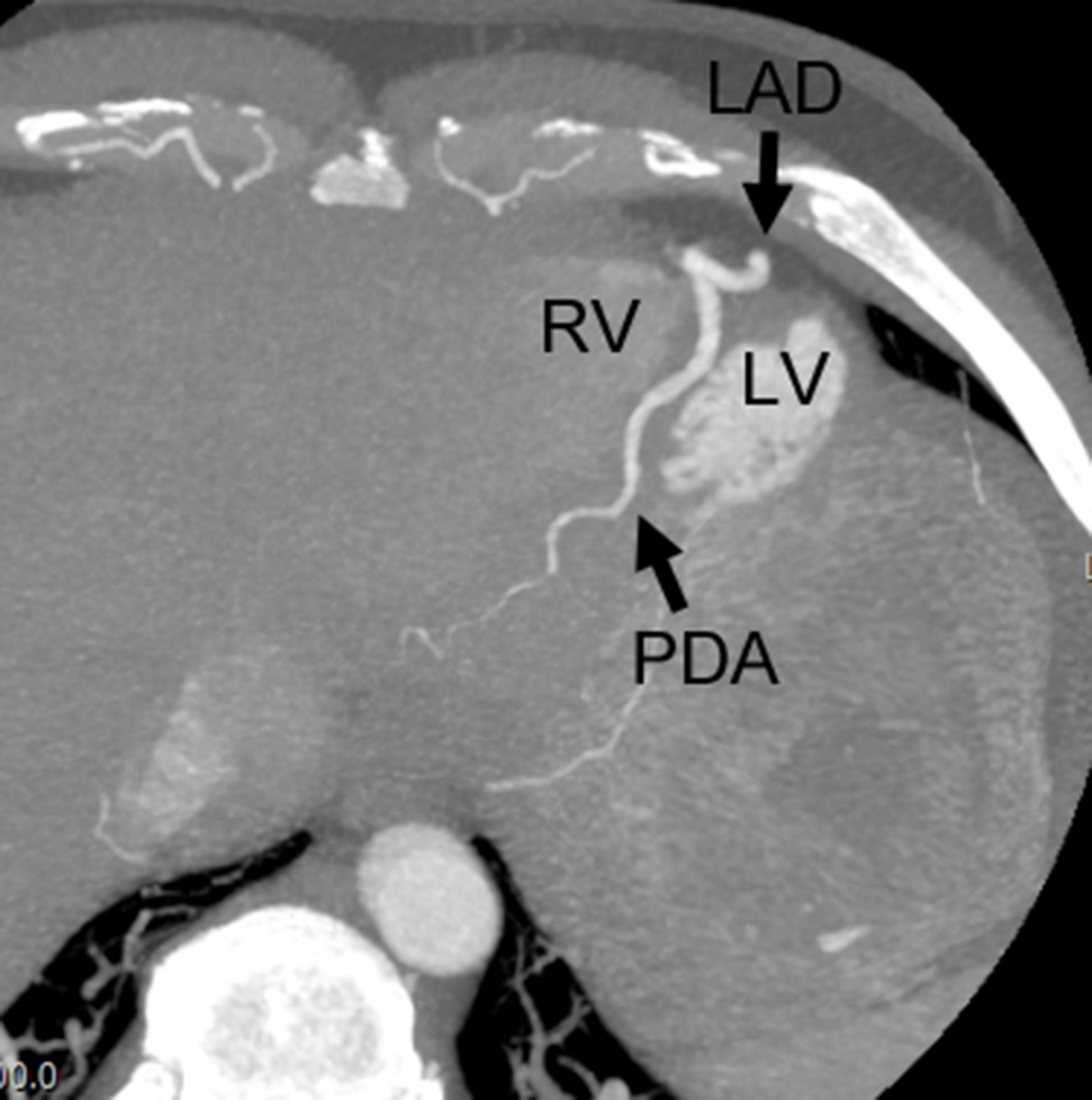
CT coronary angiogram inferior axial maximum intensity projection Figure showing inferior aspect and apex of the heart demonstrating the continuation of the left anterior descending artery (LAD) into the posterior descending artery territory (PDA), between the left ventricle (LV) and right ventricle (RV).

The CTCA showed no coronary artery disease but demonstrated an important congenital anatomical variant of the left anterior descending artery (LAD). The LAD was hyper-dominant continuing as the posterior descending artery (PDA) into the posterior interventricular groove, with a small non-dominant right coronary artery (RCA) (Figures 1–4). As the patient had not complained of any chest pain, he has been followed up in cardiology clinic and remained asymptomatic.

## Discussion

Coronary artery anomalies are rare and observed in 0.5–2% of the general population.^1^ They are often discovered incidentally using invasive or non-invasive imaging and the majority are benign with no clinical significance. A small minority however are termed to be malignant and have the potential to cause cardiac ischaemia, especially during exertion and increased myocardial oxygen demand. These malignant anomalies can be associated with symptoms including chest pain, pre-syncope, syncope, arrhythmias, myocardial infarction and even sudden cardiac death.^2^

A hyper-dominant LAD is extremely rare with only 17 cases reported in the literature.^3,4^ In a hyper-dominant LAD, the PDA arises from the LAD instead of the left circumflex artery (LCx) or RCA. The importance of this anomaly is that if a hyper-dominant LAD is occluded, it will cause a massive myocardial infarction affecting the anterior wall, septum and inferior wall. This could potentially lead to cardiogenic shock with high morbidity and mortality unless there is timely management and intervention. Some of the commonest coronary artery anomalies and their clinical significance are highlighted in the table below (Table 1).

**Table 1.** Table showing some of the commonest coronary artery anomalies, with their incidence, description and clinical significance

Coronary artery anomaly	Incidence	Clinical significance
Split RCA where it splits into an anterior and posterior branch, featuring a split posterior descending branch	<1%	Myocardial infarction would involve a large myocardial territory (both the inferior and anterior right ventricular walls)
Separate origins of the LAD and LCx	~0.3%	Benign but can be challenging to locate coronaries during coronary angiography
Ectopic RCA from the left coronary sinus	~0.3%	An interarterial subtype of this anomaly can be malignant leading to sudden cardiac death
LCx originates from the right coronary sinus	~0.2%	Usually benign
LAD originates from the right coronary sinus or from RCA	<0.1%	An interarterial subtype of this anomaly can be malignant and should be surgically fixed
Single coronary artery originating from either the left or right aortic sinus and gives rise to the entire coronary circulation	<0.1%	Can be benign although myocardial infarction or sudden cardiac death may occur in cases in which the course of the coronary artery is interarterial
All three coronaries originate from right coronary cusp	<0.1%	Usually benign
Coronary fistulae leading to abnormal communication between coronary arteries and cardiac structures or pulmonary arteries	<0.02%	Clinical presentations vary depending on the type of fistula. Patients are mostly asymptomatic but complications include myocardial ischaemia, bacterial endocarditis and heart failure

RCA, right coronary artery; LAD, left anterior descending artery; LCx, left circumflex artery

The management of anomalous coronary arteries requires careful consideration. It is essential to thoroughly evaluate patients with a physical examination and mapping of the coronary arteries. This can be done in a manner of different ways but CTCA is the state-of-the-art technique for this purpose. If a patient is identified to have a symptomatic malignant anatomy, treatment should be offered, which can be in a form of cardiac surgery.^3^ Cardiologists and cardiac surgeons should be aware of important coronary anomalies including the hyper-dominant LAD as it could have a considerable impact on clinical outcomes.

## Learning points

- Anomalous coronary arteries are rare but can be malignant and therefore cardiologists should be aware of their clinical significance.
- Occlusion of a hyper-dominant left anterior descending artery can cause a large myocardial infraction.
- CT coronary angiography is an excellent and non-invasive tool for diagnosing such abnormalities.
